# Prevalance of non-cardiac pathology on cardiovascular magnetic resonance studies

**DOI:** 10.1186/1532-429X-13-S1-P174

**Published:** 2011-02-02

**Authors:** Elisa McAlindon, Julian Strange, Nathan Manghat, Mark CK Hamilton, Peter Wilde, Peter Drivas, Dudley Pennell, Chiara Bucciarelli-Ducci

**Affiliations:** 1 CMR Unit, Bristol Heart Institute, NIHR Biomedical Research Unit, Bristol, UK; 2 CMR Unit, Royal Brompton Hospital, NIHR Biomedical Research Unit, Bristol, UK; 3 CMR Unit, Bristol Heart Institute/ CMR Unit, Royal Brompton Hospital, NIHR Biomedical Research Units, Bristol/ London, UK

## Background

Cardiovascular magnetic resonance (CMR) is routinely used in clinical practice. Although the scan focuses on the cardiovascular system, the large field of view used in the initial dataset of images includes the thorax and upper abdomen. The prevalence of incidental non-cardiac findings is not well described. This multicentre retrospective study investigates the prevalence of incidental non-cardiac pathology in clinical patients undergoing CMR.

## Methods

We reviewed consecutive CMR clinical reports of two dedicated CMR Units (Royal Brompton Hospital and Bristol Heart Institute) over a period of 3 years, from 2007 to 2010. All the scans were performed in 1.5T Avanto scanner (Siemens, Erlangen, Germany). Non-cardiac pathology was subsequently further classified in a) benign findings or b) findings requiring further characterisation. In addition, all images acquired in both centres during the month of August 2010 were retrospectively reviewed by a trained CMR cardiologist specifically assessing for non-cardiac findings.

## Results

**Figure 1:**
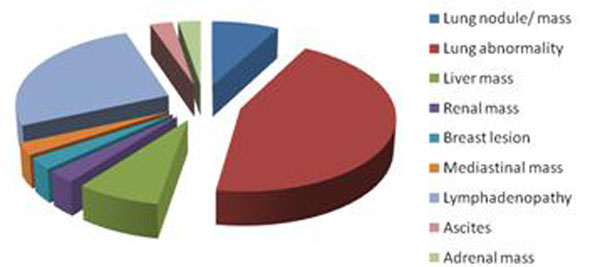
Non-cardiac findings requiring further investigation.

A total of 16,518 reports were reviewed and non cardiac pathology identified in 7.5% (n=1,242) patients. The entire image dataset of all the scans performed over a month period in both centres (n=479) were reviewed and revealed 26% (n=123) of patients with at least one non-cardiac finding (21 patients had >1 finding). A total of 144 non-cardiac pathologies were identified, of which 106 were classified as benign findings and 38 as findings requiring further characterisation and testing (Figure 1). In particular, 10/38 lesions identified (2% of patients) represented possible malignancies (in particular mediastinal and lung lesions).

## Conclusions

Whilst the prevalence of incidental findings by reports review (7.5%) is in keeping with the previous small single centre reports, this appears to underestimate the true incidence of non-cardiac pathology. Retrospective image review identified a higher prevalence of non-cardiac pathology (26%) which is similar to previously reported incidence during cardiac computed tomography. In our study, 8% of the patients required further testing following CMR and 2% of the patients had possible malignancies. Follow-up is required to establish the clinical significance of these findings and their impact on patient management.

